# Non-Invasive Sheep Biometrics Obtained by Computer Vision Algorithms and Machine Learning Modeling Using Integrated Visible/Infrared Thermal Cameras

**DOI:** 10.3390/s20216334

**Published:** 2020-11-06

**Authors:** Sigfredo Fuentes, Claudia Gonzalez Viejo, Surinder S. Chauhan, Aleena Joy, Eden Tongson, Frank R. Dunshea

**Affiliations:** 1 Digital Agriculture, Food and Wine Sciences Group, School of Agriculture and Food, Faculty of Veterinary and Agricultural Sciences, The University of Melbourne, Parkville, VIC 3010, Australia; cgonzalez2@unimelb.edu.au (C.G.V.); eden.tongson@unimelb.edu.au (E.T.); 2 Animal Nutrition and Physiology, Faculty of Veterinary and Agricultural Sciences, The University of Melbourne, Parkville 3010, Australia; ss.chauhan@unimelb.edu.au (S.S.C.); aleenajoyj@student.unimelb.edu.au (A.J.); fdunshea@unimelb.edu.au (F.R.D.); 3 Faculty of Biological Sciences, The University of Leeds, Leeds LS2 9JT, UK

**Keywords:** animal welfare, skin temperature, artificial intelligence, heart rate, respiration rate

## Abstract

Live sheep export has become a public concern. This study aimed to test a non-contact biometric system based on artificial intelligence to assess heat stress of sheep to be potentially used as automated animal welfare assessment in farms and while in transport. Skin temperature (°C) from head features were extracted from infrared thermal videos (IRTV) using automated tracking algorithms. Two parameter engineering procedures from RGB videos were performed to assess Heart Rate (HR) in beats per minute (BPM) and respiration rate (RR) in breaths per minute (BrPM): (i) using changes in luminosity of the green (G) channel and (ii) changes in the green to red (a) from the CIELAB color scale. A supervised machine learning (ML) classification model was developed using raw RR parameters as inputs to classify cutoff frequencies for low, medium, and high respiration rate (Model 1). A supervised ML regression model was developed using raw HR and RR parameters from Model 1 (Model 2). Results showed that Models 1 and 2 were highly accurate in the estimation of RR frequency level with 96% overall accuracy (Model 1), and HR and RR with R = 0.94 and slope = 0.76 (Model 2) without statistical signs of overfitting

## 1. Introduction

Live animal exports have been lately under scrutiny by the public and animal welfare advocates [1], especially live export though shipping, related to welfare conditions and heat stress during long trips up to six weeks by sea, which in extreme cases can result in the death of animals in rates up to 2–3.8% [2]. Specifically, these mortality rates have been recently found in animal shipments from Australia through the Persian Gulf, which can reach temperatures of 36 °C with 95% relative humidity resulting in heat stress [3].

Heat stress events for animals are not only restricted to animal transport through sea or land, but it can also happen in farms due to increased ambient temperatures related to climate change, which can directly impact the health and welfare of animals [4,5,6,7]. There have been several types of research investigating the genetic resilience and adaptation of animals to heat stress [8,9,10,11] and mitigation strategies [12,13,14]. Many of these studies have based their assessment of heat stress on environmental indices, such as ambient temperature and relative humidity combined to form a temperature-humidity index (THI) [14,15,16]. The THI can be coupled with direct assessment of the effects of heat stress using physiological responses through manual monitoring [17,18], using sensors directly located on animals [19], behavioral assessments or including molecular, cellular and metabolic biomarkers [20,21,22]. These methods, though very reliable and robust, are intensive, requiring animal restraining, are labor-intensive, and time-consuming, also requiring specialized instrumentation and technical know-how from the personnel acquiring the data. Moreover, the use of intravaginal/rectal devices or contact sensors can be stressful for the animals [23].

Applications of artificial intelligence (AI) and machine learning modeling (ML) have been recently implemented to analyze environmental factors, such as THI, and its effects on heat stress of dairy cows and final productivity and quality of milk to maximize the utility of big data available from robotic dairy farms [24]. Further, AI and ML have been applied for processing and modeling remotely sensed information, which may offer a powerful tool to automatically extract critical physiological data from videos and infrared thermal imagery from animals and welfare analysis or the effects on quality of products [23,25,26,27]. 

Non-invasive methods to assess heat stress, based on remote sensing, have shown to be promising, as they avoid biases in the physiological data obtained from animals due to stresses imposed by wearable sensors, such as collars, polar sensors (for respiration and heart rate measurements), or intravaginal/rectal sensors for body temperature measurements [23,28]. Specifically, computer vision and infrared thermal remote sensing techniques have been recently applied to assess animal stress based on skin temperature and respiration rate [23,29] or the detection of heart rate and respiratory rates in pigs through luminosity changes from RGB videos of animals [29,30]. 

One of the main constraints in applying remote sensing techniques on sheep involves the thick fleece from unshorn animals, which presents a thick resistance layer from the skin. The advantage of utilizing these remote sensing techniques on pigs, especially hairless breeds, is that reflectance from visible and infrared thermal wavelengths are a direct representation of skin changes. Hence, non-invasive methods are required to be applied to body sections with less hair or wool in sheep and with outputs that can be representative, such as the head and face parts [23,30,31]. Specifically, these areas mainly correspond to the nose for respiration and heart rate and the whole head for skin temperature, especially focused on the eye section, since they are the only exposed internal organs to the environment, which may represent core body temperatures.

This study aimed to test a non-contact biometric system based on artificial intelligence to assess heat stress of sheep to be potentially used as automated animal welfare assessment in farm and while in transport. Specifically, it was focused on the automatic tracking of regions of interest (ROI) from sheep RGB videos and infrared thermal videos (IRTV) and the assessment of physiological information such as skin temperature, respiration rate (RR), and heart rate (HR) modeled using machine learning algorithms of sheep subjected to thermoneutral and controlled heat stress conditions. The system proposed was based on an affordable and integrated RGB 4K video camera and a high-resolution thermal infrared camera. It was further recommended an artificial intelligence approach to extract information automatically from sheep that could be coupled to blockchain [32,33] to have an independent assessment of animal welfare to be applied in the farm and transport or vessel environments. The latter could allow research on automated systems to ameliorate heat stress on farm animals or during transportation, such as mister or sprinklers, and will offer a blockchain system for control and certification of good practices on the farm or transportation to abattoirs or export markets.

## 2. Materials and Methods

### 2.1. Location, Animal Treatments, and Data Acquisition

**Figure 1:**
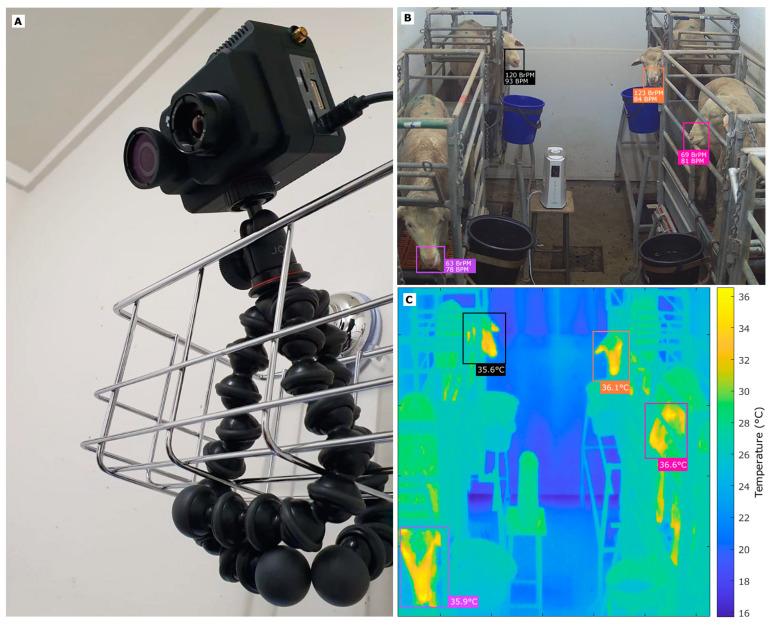
Images showing the experimental layout for thermoneutral (control) and heat stress chambers and implementation of feature tracking algorithms and machine learning (ML) Models 1 and 2 developed, with (**A**) the FLIR^®^ Dup Pro camera setup, (**B**) shows the selected region of interest (ROI: nose) from each sheep visible and extraction of corresponding respiration rate and heart rate values from the video analysis using machine learning (Models 1 and 2), and (**C**) the selected region of interest (ROI: face) from each sheep and automatic tracking and extraction of temperature values (°C) from the infrared thermal video (IRTV) analysis. Abbreviations: BrPM: breaths per minute; BPM: beats per minute.

This study was based on live animals and approved by the Faculty of Veterinary and Agricultural Sciences, University of Melbourne Animal Ethics Committee (AEC#1914872.1). It was conducted at The University of Melbourne (UoM), Dookie Campus, Victoria, Australia (36°22′48″ S, 145°42′36″ E). Twelve sheep (Merino lambs 4–5 months old) were acclimatized to indoor facilities and housed in the individual pens for 3 days before starting measurements. They were fed a mixed ration (50% pellets, 25% oaten, and 25% Lucerne chaff) formulated to meet or exceed the National Research Council (NRC) [34] requirements, complemented with fresh water ad libitum. Room exhaust ventilation was performed using fans through the whole time of the experiments to simulate ventilation usually performed during live sheep export shipments. The latter mainly rely on mechanical ventilation using fans to remove heat and water vapor produced by animals, to ventilate moisture produced from manure pads, and to remove any possible build-up of noxious gases. After acclimatization, sheep were relocated to metabolic cages and housed in two temperature and relative humidity control rooms (Figure 1), conditioned to have two treatments with six sheep exposed to cyclic heat stress: (i) room at 28–40 °C and 40–60% relative humidity (RH), the cycles consisted of high temperatures of 36–40 °C every day from 8:00 to 16:00, and then reduced to 28–30 °C, and thermoneutral (control) conditions (ii) room at 18–21 °C and RH between 40 and 50%. The temperature (*T*) and RH were recorded every 30 min in each room using a universal serial bus (USB) temperature and humidity data logger (TechBrands; Electus Distribution, Rydalmere, NSW, Australia). These data were used to calculate the *THI* using the formula from Equation (1), which was specially developed for sheep [35].

(1) THI=T−⌈(0.31−0.31RH)(T−14.4)⌉

An integrated RGB video and infrared thermal video (IRTV) camera, FLIR^®^ Duo Pro (FLIR Systems, Wilsonville, OR, USA) was fixed in each room using a small rack and tripod for stabilization (Figure 1A). This device has two cameras to record simultaneously RGB videos (Resolution: 4000 × 3000; Field of View: 56° × 45°) and IRTV (with a resolution of 336 × 256; Field of View: 35° × 27°; Thermal Sensitivity: <50 mK; Thermal Frame Rate: 9 Hz; Accuracy: ±5 °C). The camera has Bluetooth^®^ connectivity (Bluetooth Special Interest Group, Kirkland, WA, USA) and, hence, can be controlled remotely using a FLIR smartphone/tablet personal computer (PC) application, FLIR^®^ UAS 2 (FLIR Systems, Wilsonville, OR, USA). The RGB video and IRTV data were recorded three times daily (8:00; 12:00; 16:00) during 1 min each time for four weeks to have a wider range of physiological data. 

Two kinds of measurements were conducted using: (i) traditional/manual techniques, and (ii) non-contact biometrics based on remote sensing (Figure 1B). The manual methods consisted of (i) heart rate (HR) using an elitecare^®^ Sprague stethoscope (eNurse, Brisbane, QLD, Australia) and a timer, (ii) respiration rate (RR) visually with a chronometer assessing animal inhalations and exhalations, (iii) skin temperature from the right flank, below the wool in contact with the skin using a digital thermometer (Model: DT-K11A; Honsun, Shanghai, China), and (iv) rectal temperature using the same type of digital thermometer (Model: DT-K11A). The remotely sensed data (FLIR camera) were recorded using two FLIR^®^ Duo integrated cameras for 1 min on each side of the room to capture all sheep three times a day, as previously mentioned. The thermal videos were used to assess skin temperature. In contrast, the RGB videos were recorded to evaluate HR and RR using computer vision analysis and customized ML modeling developed by the Digital Agriculture Food and Wine Group (DAFW) from UoM based on changes in luminosity within the RGB (HR) and Lab (RR) channels that have been developed for humans and animals based on the photoplethysmography (PPG) principle [23,30,31,36,37,38]. For the validation/calibration purposes of these newly developed ML models, only the most representative recordings were used; therefore, not all sheep were analyzed due to chamber size restrictions.

### 2.2. Computer Vision Analysis to Obtain Biometrics

The radiometric IRTVs were saved in sequence file extension (seq) and batch converted to Audio Video Interleaved (AVI) using the Sense Batch software (Sense Software, Warszawa, Mazowsze, Poland). The latter was also used to extract in batch and parallel the radiometric data from each frame from all thermal videos in comma-separated values (csv) files. The IRTV was imported to MATLAB^®^ R2020a (MathWorks Inc., Natick, MA, USA) and the Video Labeler functions from the Computer Vision Toolbox™ 9.2 in MATLAB^®^ R2020a were then used to select and track ROIs focusing on the head from each animal (automatic). Specifically, for sheep, the face was selected because the hottest visible spots are found in the eyes and nose (Figure 1C). Once the ROIs were tracked, labels were saved automatically, and a customized algorithm written in MATLAB^®^ R2020a by the DAFW Group from UoM was used to obtain the maximum (Max), mode, and standard deviation (SD) of the temperatures from each frame from the selected ROI. Additionally, the mean, Max, mode, and SD from the Max temperatures from all frames were calculated.

For the analysis of raw signals related to HR and RR, the RGB videos acquired in QuickTime Movie (MOV) file-extension were used. These were analyzed using the Video Labeler functions from the Computer Vision Toolbox™ 9.2 in MATLAB^®^ R2020a and the point tracker algorithm, which can detect features defined as a region of interest (ROI) and track one or more region of interest (ROI) based on the Kanade–Lucas–Tomasi (KLT) algorithm. For this specific study, the nose section was used as ROI for both HR and RR analysis (Figure 1B), as this is the area in which less wool may be found, as other areas may interfere with the readings creating biases in the data extracted. The ROI labels obtained were automatically exported and used to crop the RGB videos to get smaller videos only from the nose area from each sheep selected. These cropped videos were then automatically analyzed to obtain the signal changes from luminosity and different channels (RGB, CIELAB) using a modified version of the raw video analysis (RVA) algorithm developed as a function to measure HR in humans using the photoplethysmography (PPG) method [38] developed by the DAFW Group from UoM. This algorithm applies a fast Fourier transformation (FFT) for the transformation of the time signal to frequency and uses a second-order Butterworth filter with cutoff frequencies (Hz) for analysis. To assess raw signals related to RR, the RVA algorithm was modified (RVAm) to determine the luminosity changes in the “a” channel from the CIELAB color scale (green to red). The raw signals from computer vision analysis of cropped videos were evaluated within the cutoff frequency range 0.33–3.1 Hz for a ML classification model (Model 1, detailed in the machine learning modeling subsection), and the respiration cutoff frequency ranges used were according to the outputs of Model 1 described in detail below for low: 0.2–1.2 Hz; medium: 1.2–2.2 Hz, and high: 2.2–3.2 Hz. On the other hand, to assess HR, the luminosity changes in the green (G) channel of the RGB color scale were used within a frequency range of 0.83–3.00 Hz, since the normal and stressed HR for sheep had a lower spread in values compared to RR.

**Figure 2:**
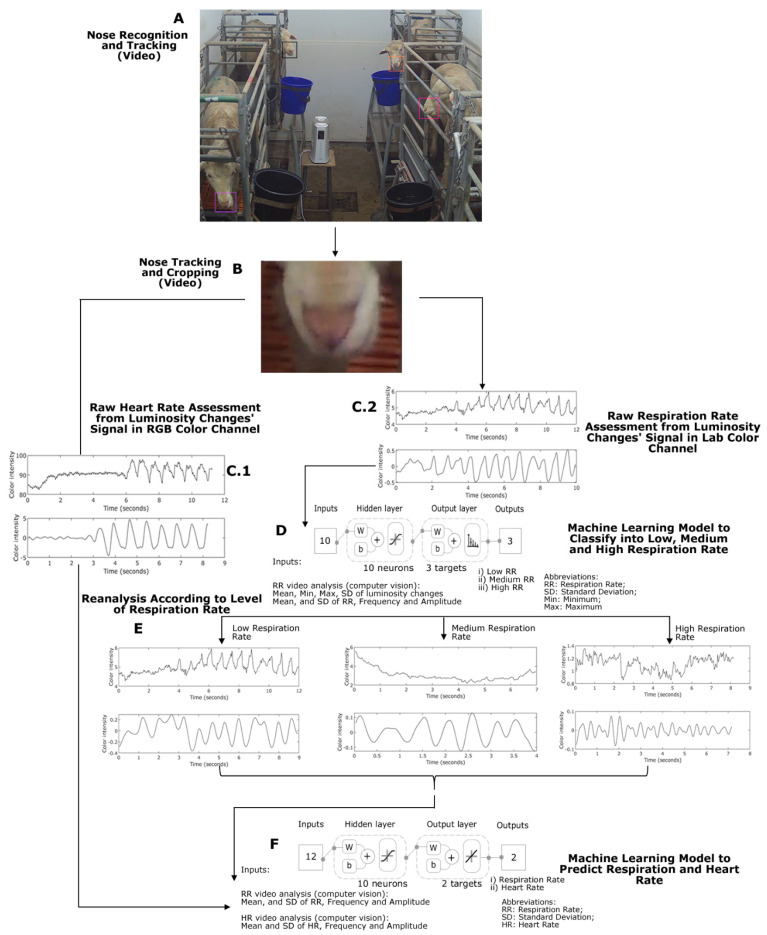
Diagram showing the algorithms pipeline for RGB video analysis process from (**A**) region of interest selection; (**B**) cropped videos from sheep feature to be analyzed; (**C1**) raw video analysis (RVA) for heart rate (HR) signals using the green channel (RGB); (**C2**) modified RVA (RVAm) for respiration rate (RR) analysis using the “a” channel (CIELAB) wide cutoff frequency range; (**D**) machine learning pattern recognition (Model 1) to obtain actual cutoff frequency range; (**E**) re-analysis of RR signals; (**F**) regression machine learning (Model 1) to obtain accurate HR an RR. Model diagram abbreviations: w: weights; b: bias.

All the steps mentioned above were automated into a pipeline code using components as functions, which are represented in the diagram of Figure 2, in which the only supervised processes are the initial ROI selection for the IRTVs and RGB Videos.

### 2.3. Statistical Analysis and Machine Learning Modeling

Linear regression analysis for temperature data with intercept passing through the origin and *p* ≤ 0.05 as criteria were used to compare the skin and rectal temperature measurements using the manual methods against each other and the non-invasive infrared thermal biometrics (IRTV) with XLSTAT ver. 2020.3.1 (Addinsoft, New York, NY, USA). Furthermore, linear regression analysis for RR and HR data measured manually and from videos using computer vision analysis with a single frequency range for RR and using frequency ranges for low, medium, and high, as previously mentioned, were performed. Statistical parameters, such as determination coefficient (R^2^), *p*-value, and root means squared error (RMSE) were calculated to test the goodness of fits.

Based on a proposed parameter engineering procedure, raw RR-related parameters obtained from RGB Video analysis, using the RVAm algorithm and a single frequency range (0.33–3.1 Hz), of mean, minimum (Min), maximum (Max), and standard deviation (SD) of luminosity changes and mean, SD, frequency, and amplitude were used as inputs to develop an initial ML supervised pattern recognition model to classify the sheep cropped videos into low, medium, and high respiration frequencies (Model 1; Figure 2D). For this procedure, a customized MATLAB^®^ code, developed by the authors, was used to test 17 artificial neural networks (ANN) training algorithms [39]. The Bayesian Regularization algorithm was selected as the best performing algorithm from this procedure based on the accuracy [correlation coefficient (R)] and best performance (means squared error (MSE)) with no signs of overfitting. This algorithm does not require a validation stage as it updates the weights and biases according to the optimization of the model, and is very effective on avoiding overfitting especially for small and/or noisy datasets [39,40,41]. Samples were divided randomly with 70% used for training (*n* = 94), and 30% for testing (*n* = 40). Figure 2D shows the model diagram with the two-layer feedforward network with a tan-sigmoid function in the hidden layer and Softmax function in the output layer. Ten neurons were selected as the best performance with no under- or over-fitting, which was obtained from a neuron trimming test (data not shown). 

Once the videos of sheep were classified automatically into low, medium, and high RR by cutoff frequency ML analysis, the videos are automatically reanalyzed using the corresponding frequency ranges, low: 0.2–1.2 Hz; medium: = 1.2–2.2 Hz; high: 2.2–3.2 Hz, by calling three separated functions. From this analysis, the outputs from raw RR and HR parameters were used as inputs to develop a fitting/regression model to predict the real values of RR and HR based on the manual measurements as targets (Model 2). Again, 17 different training algorithms [40] for artificial neural networks (ANN) were assessed in batch to find the best model based on output statistics. The Bayesian Regularization algorithm was selected as the best performing from this procedure. For modeling purposes, samples were divided randomly as follows: 70% (*n* = 94; observations (*n* × targets) = 188) for training and 30% (*n* = 40; observations (*n* × targets) = 80) for testing. Figure 2F depicts the model diagram showing the two-layer feedforward network with a tan-sigmoid function in the hidden layer and a linear transfer function in the output layer. Ten neurons were selected as the best performance with no under- or over-fitting, which was obtained from a neuron trimming test (data not shown). 

Multivariate data analysis based on a biplot (variables and samples) of principal component analysis (PCA) was performed using XLSTAT to find relationships and patterns among the data between real physiological parameters and estimated using computer vision tools and models proposed. The cutoff point of 60% of data variability explained by the total of both PC1 and PC2 was considered to test significance [42]. The THI index calculated using Equation (1) was also included to compare data from sheep in control and heated chambers.

## 3. Results

**Figure 3:**
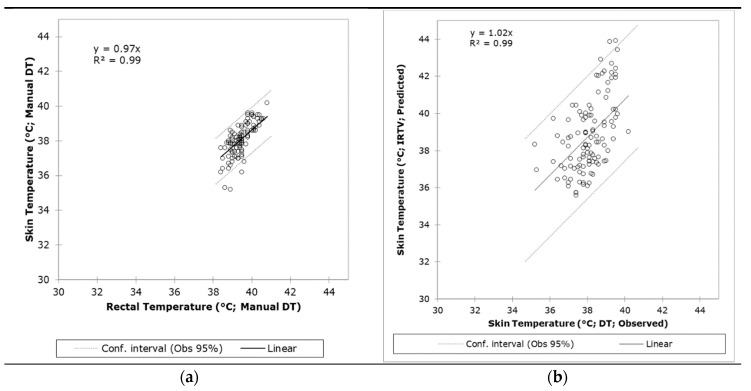

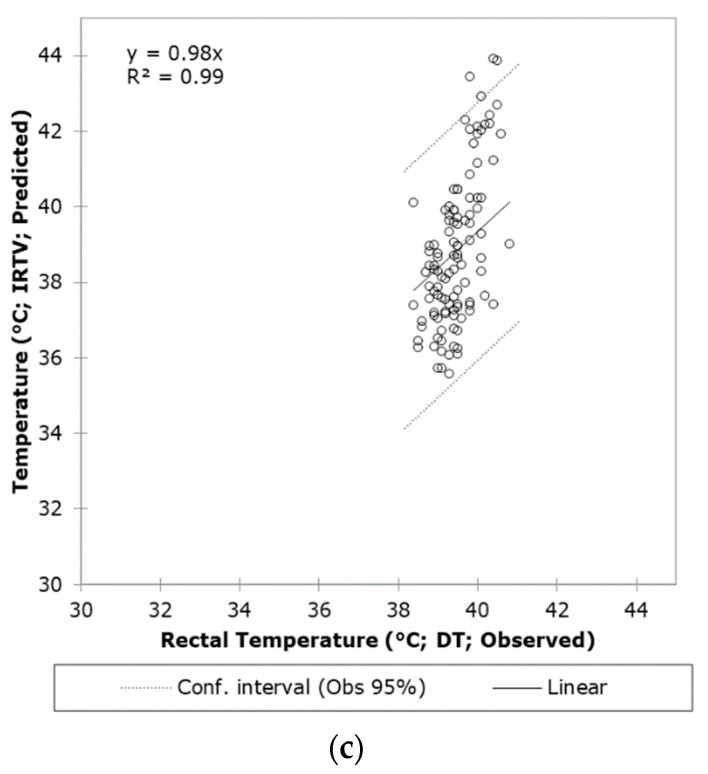
Linear regressions comparing results from (**a**) rectal vs. skin temperatures measured manually using a digital thermometer (DT), (**b**) observed skin temperature (manual) vs. temperature from the infrared thermal video analysis (IRTV), and (**c**) observed rectal temperature (manual) vs. temperature from the IRTV analysis. Abbreviations: Obs: observed, Conf: Confidence.

Figure 3a shows the results from the linear regression of rectal and skin temperatures measured with the manual/traditional methods compared to those obtained from the IRTV analysis. There was a narrow distribution of temperatures from all sources (from around 35−40 °C) since the study was performed on live animals. The linear regression passing through the origin (0,0) was statistically significant (*p* < 0.001) and presented a very high correlation and determination coefficients (R = 0.99; R^2^ = 0.99; RMSE = 0.66; slope =0.97) between these two parameters with 3.6% of outliers (4 out of 110) based on the 95% confidence intervals. On the other hand, Figure 3b shows the results from the linear regression of observed skin temperature (manual/traditional methods) and the values obtained from the remote sensing analysis using the IRTVs. These relationships were also statistically significant (*p* < 0.001) with an R^2^ = 0.99 (R = 0.99); RMSE = 1.66; slope = 1.02. Based on the 95% confidence intervals, it only had 2.73% of outliers (3 out of 110). Similarly, Figure 3c shows the results from the linear regression of observed rectal temperature (manual/traditional methods) and the values obtained from the remote sensing analysis using the IRTVs. The lineal model resulted with very high correlation and determination coefficients (R = 0.99; R^2^ = 0.99) and was statistically significant (*p* < 0.001) with RMSE = 1.71; slope = 0.98 and 3.6% of outliers (4 out of 110) based on the 95% confidence intervals.

**Figure 4:**
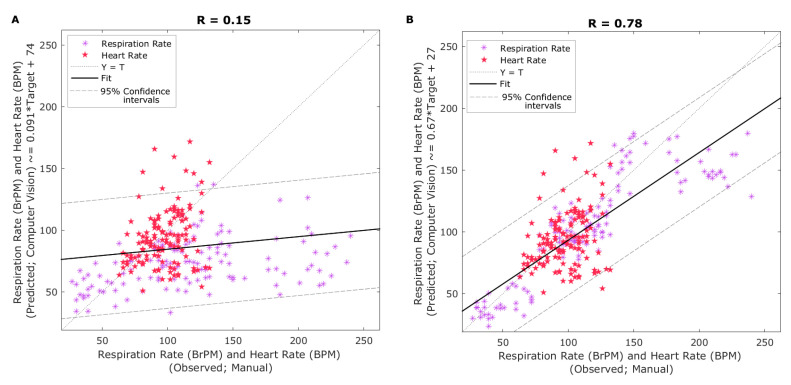
Linear regressions comparing results from (**A**) respiration rate (RR) and heart rate (HR) measured manually (x-axis) and using computer vision analysis with a single cutoff frequency range for respiration rate (0.33–3.1 Hz; y-axis), and (**B**) respiration rate and heart rate measured manually (x-axis) and using computer vision analysis with the corresponding cutoff frequency range for respiration rate according to low, medium or high respiration rate (low: 0.2–1.2 Hz; medium: = 1.2–2.2 Hz; high: 2.2–3.2 Hz; y-axis).

Figure 4A shows the linear regression between RR and HR measured manually and raw signal analysis related to HR and RR using computer vision analysis with a single cutoff frequency range for respiration rate (0.33–31. Hz) and HR (0.83–3.00 Hz). It can be observed that the correlation and determination coefficients were very low (R = 0.15; R^2^ = 0.02; *p* < 0.001) with RMSE = 23.73 and slope = 0.09 mainly represented by the poor correlation found for the RR raw data. On the other hand, Figure 4B shows the same manually measured HR and RR rates against the raw computer vision analysis using different cutoff frequency ranges for RR according to low, medium, or high values (low: 0.2–1.2 Hz; medium: = 1.2–2.2 Hz; high: 2.2–3.2 Hz). It can be observed that the correlation increased significantly (R = 0.78; R^2^ = 0.61; *p* < 0.001) compared to Figure 4A, with RMSE = 21.81 and slope = 0.67.

**Figure 5:**
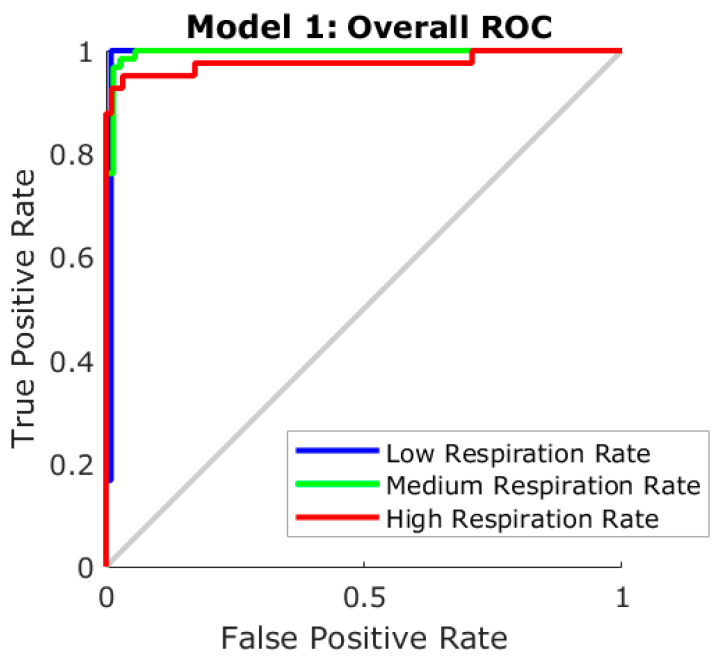
Receiver operating characteristics (ROC) curve of the pattern recognition (Model 1) depicting the true-positive and false-positive rates.

Table 1 shows the results from the ML pattern recognition model to classify cropped videos from sheep into low, medium, and high RR, according to the cutoff frequencies for these three levels. It can be observed that it presented a very high overall accuracy (96%) with no signs of overfitting as the MSE of the training stage (MSE < 0.01) was lower than the testing (MSE = 0.10). Figure 5 depicts the receiver operating characteristics (ROC) curve with the true positive (sensitivity) and false-positive (specificity) rates of the three categories, with all three categories within the true positive side of the curve; the high RR group presented the lowest sensitivity.

**Figure 6:**
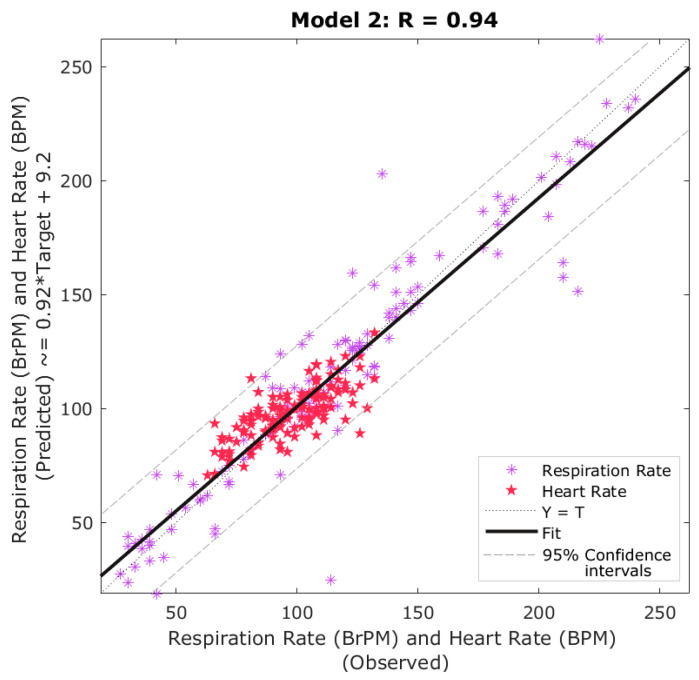
Artificial neural network overall fitting model showing the correlation coefficient (R), observed (x-axis), and predicted (y-axis) respiration rate and heart rate values. Abbreviations: BrPM: breaths per minute; BPM: beats per minute; T: Targets.

Table 2 shows the results of the ML model developed using the results from the remote sensing analysis proposed to obtain physiological parameters using RGB video, computer vision, and ML modeling (Figure 2) to extract parameters used as inputs to predict RR and RH. It can be observed that the overall model presented a high correlation (R = 0.94) and slope close to the unity (0.92) with no signs of overfitting as the performance MSE value of the training stage (MSE = 72) was lower than the testing (MSE = 512). Furthermore, the overall model presented 9.7% of outliers (13 out of 134) based on the 95 confidence intervals (Figure 6).

**Figure 7:**
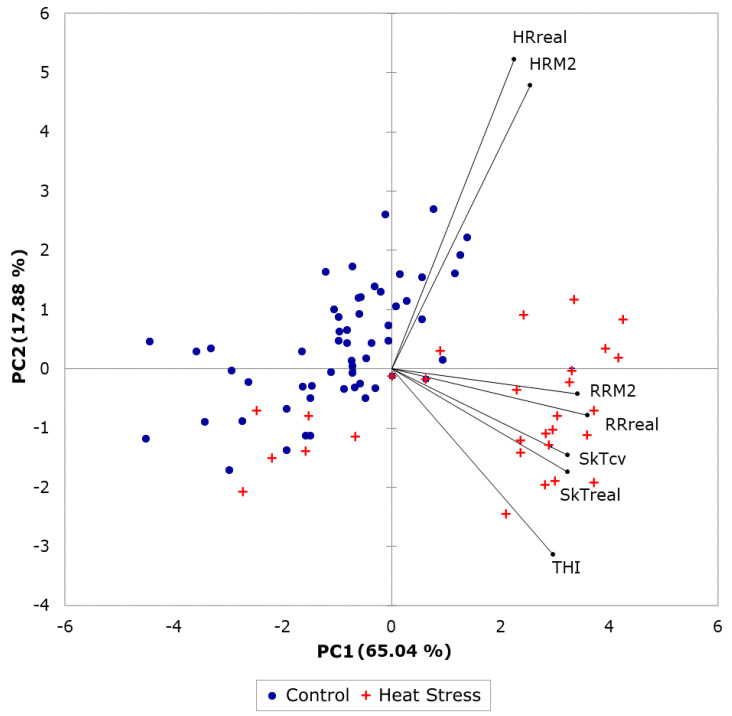
Principal components analysis of data measured with manual techniques (i) SkTreal: skin temperature real, (ii) HRreal: heart rate real, (iii) RRreal: respiration rate real, and those measured using computer vision analysis and predicted using the machine learning models (iv) SkTcv: skin temperature computer vision, (v) RRM2: respiration rate from Model 2, (vi) HRM2: heart rate from Model 2, and the temperature-humidity index (THI) from each day of the control and heat stress rooms.

Figure 7 shows the PCA comparing the HR, RR, and skin temperature measured with manual techniques (HRreal, RRreal, and SkTreal) with those predicted using Model 2 (HRM2, and RRM2) and measured by computer vision algorithms (SkTcv), as well as the THI (Equation (1)) for sheep from both treatments (control and heat stress) in different days/times of measurements. The resulting PCA described a total of 82.92% of total data variability (PC1: 65.04%; PC2: 17.88%). It can be observed that the manual measurements and those assessed using the proposed methods were closely related. Furthermore, skin temperature was related to THI. As expected, there was a clear separation and clustering between the sheep physiology under control treatment (blue circles) compared to those under heat stress (red crosses), with the latter associated with higher RR, skin temperatures, and THI. PC1, which is the main responsible for the separation of the data according to the treatments, is more related to RR and skin temperature, with HR with lower variability related to PC2. The THI values obtained in this study ranged from 18 to 20 for control and between 27 and 36 for heat stress conditions (data not shown).

## 4. Discussion

### 4.1. Selection of Critical Sheep ROIs, Features Tracking, and Automation

**Figure 8:**
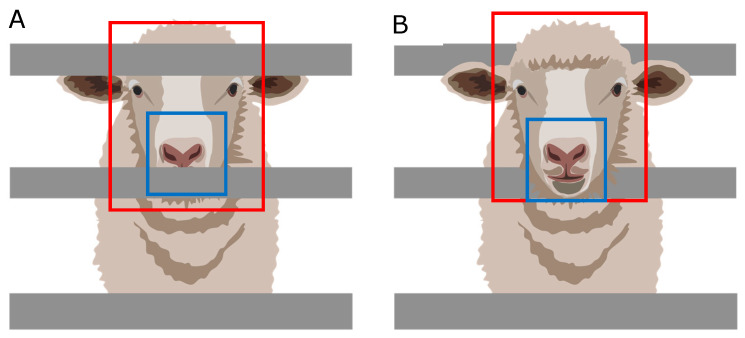
Examples of sheep with head and features tracked as region of interest (ROI) for the head (blue rectangles) for infrared thermal video (IRTV) analysis and nose/mouth regions (blue rectangles) for heart and respiration rates analysis from RGB video analysis behind bars (**A**) and through the bars (**B**) of cages.

Due to constraints in the experimental chambers related to interference from the metabolic cages and sheep head movement through them, especially while feeding (Figure 8), it was not possible to use single co-registered ROIs for RGB videos and IRTV. The latter would have simplified the modeling procedure; however, results could have been only applicable to animals with visible features and with no obstructions through the whole video recordings, rather than those with obstructions like bars from the cage (Figure 8A,B). Hence, the methodology proposed has greater practical applications in penned and transported animals. By selecting the whole head of the sheep as ROI for IRTV analysis (Figure 8; red rectangles), with automated maximum temperature extraction, it gives a higher probability of extracting meaningful temperature information from the eyes, nose or mouth regions at any specific time from cropped videos even when the head moves across obstacles, such as bars from the cages (Figure 8). Furthermore, without obstructions and considering nose and mouth regions as ROI, a simplified skin temperature extraction could also have been easily implemented by signal analysis of peaks and valleys that represents mathematically the variability between temperatures related to inhalations and exhalations using a similar RVA analysis of the signal (Figure 2). However, considering obstacles (metabolism cage bars) and head movement through them (Figure 8), the temperature variability could have been biased and difficult to discriminate from those related to obstacles (Figure 8A), which would have rendered this potential simple procedure with higher errors in skin temperature estimation similar to Figure 4A. On the contrary, the method proposed in this research resulted in high accuracy compared to measured skin and rectal temperatures with closer relationship from the 1:1 line for skin temperatures as expected (Figure 3).

In the case of HR and RR, ROIs were selected from the nose/mouth region of the animals since they have more hairless skin exposed (Figure 8; blue rectangles). The nose region is considered the best area to measure RR as it is where inhalation and exhalation occurs and the area in which a large number of blood vessels are found in sheep [43,44], which also allows measuring HR more accurately. Specifically, from these ROIs, changes in luminosity are related to the rushing in and out of the bloodstream and cooling down and warming up of skin surfaces, which can be related to HR and RR, respectively. This would happen obviously on surfaces of living and breathing organisms, making inanimate obstacle’s luminosity unchanged and easy to discriminate either by signal analysis from RGB video or to be detected by ML modeling.

### 4.2. Computer Vision Analysis of Raw Signals Obtained from Videos

The sensitivity of the raw signal analysis extracted from computer vision algorithms related to raw HR and RR can be determined by the use of specific cutoff frequency ranges (Hz) as determined by the RVA and RVAm algorithms [38]. Since the range of RR observed for sheep under control and heated environments is very high (27–240 BrPM) compared to HR (63–132 BPM), the selection of cutoff frequency to extract RR raw data is critical. By selecting the whole range cutoff frequency range, results showed low sensitivity when compared to observed RR data (Figure 4A).

Accuracies for HR and respiration rates found through computer vision analysis with different cutoff frequencies for sheep to obtain raw HR and RR (Figure 4B) were in accordance to those using similar methodologies for cattle [31] and pigs [30]. In the case of HR, this study had a narrower range between 55 and 135 BPM, with the lower range consistent to the average HR reported for lambs without stress of 57 ± 5 [45]. 

For RR rate analysis, a review showed that by using cutoff frequencies between 0.20 and 0.40 Hz in the case of sheep and goats corresponded to RR of 12–24 BrPM. However, a breathing frequency study in ruminants recorded RR of 54 BrPM, equivalent to 0.9 Hz, which is within the reference range for adult sheep of 12–72 breaths per minute [46]. In this study, RR ranged from around 45–260 BrPM, which is consistent with ranges found in other sheep studies under normal and heat stress conditions, such as BrPM values between 31 and 247 BrPM [47]. Hence, higher RR corresponding to stressed sheep corresponded to three times higher than those associated with around 1 Hz frequency. By using maximum values of 3 Hz for higher RR values, resulting in more accurate raw RR obtained from computer vision algorithms (Figure 4B).

### 4.3. Machine Learning Modeling to Extract Further Sheep Biometrics

The ANN pattern recognition algorithm (Model 1) was able to pre-process the data to obtain these specific cutoff frequency ranges for RR analysis, which increased the accuracy and performance of Model 2 compared to lower accuracies and performances using a single frequency cutoff range for both HR and RR and computer vision analysis (Figure 4B). By using computer vision analysis for skin temperature extraction and ANN models 1 and 2 allowed full automation in the estimation of HR and RR from RGB video and IRTV. Furthermore, the integrated FLIR cameras used have direct connectivity drivers to be used within MATLAB^®^ environments that can allow real-time extraction of sheep biometrics using the codes developed in this study as shown in Figure 1B, C. From Figure 2, the only supervised procedure is the initial ROIs selection for visible animals, making the rest of the process automatic through the pipeline of algorithms, functions and ML models proposed (Figure 2).

Pre-processing of videos using a wider cutoff frequency range for signal analysis of cropped videos from sheep, plus classification (Model 1) and re-analysis, it takes around 20 s for a 1-min video approximately, using parallel computing capabilities on a 4-core laptop PC. Hence, using higher computer capabilities, it was estimated for this time requirement to be between 3–5 s to allow signal stabilization and cut start to initiate real-time rendering of outputs as shown in Figure 1B, C. Specifically for HR in humans, the same pre-analysis periods can be found for commercial software, such as FaceReader (Noldus, Wageningen, The Netherlands) and the computer application Cardiio (Cardiio, Inc., Cambridge, MA, USA) for smartphones and tablet PCs.

### 4.4. Comparison between Non-Invasive Biometrics and Environmental Heat Stress Indices

In previous studies, sheep exhibit heat stress with THI ≥ 23 for Mediterranean dairy sheep and THI ≥ 27 in Comisana dairy sheep [48]. These THI values are consistent with the ranges for heat stress treatment applied in this study (THI: 26–36). Sheep regulate heat through panting mainly; hence, the RR is the main heat regulatory mechanism for these animals [47]. Very high RR values found for heat-stressed sheep (260 BrPM) were related to the highest THIs between 27 and 36 (Figure 7). From the same figure, vectors related to THI, skin temperature, and RR (for both observed and extracted through biometrics) were related as expected, which help to maintain a relatively constant maximum HR showing heat stress regulation. Higher THI is related to higher panting from sheep, which helps reducing skin and the internal temperature of animals. Some sheep from the heat stress treatments appear in the PCA graph close to the control cluster, which may correspond to more genetically resilient sheep to heat stress [49,50].

### 4.5. Artificial Intelligence System Proposed Based on Algorithms and Models Developed

Automatic ROI selection from sheep can be achieved through the training of deep learning algorithms to recognize specific sheep features, such as those for the head and nose/mouth regions. This has been achieved using convolutional networks for pigs [51], wildlife animals [52], marine animals [53], and chimpanzee faces [54], among others. Other methodologies based on ML, such as discriminant analysis, independent components Analysis, and ANN, among others, have been used for animal detection, classification, and tracking [55,56,57,58,59].

**Figure 9:**
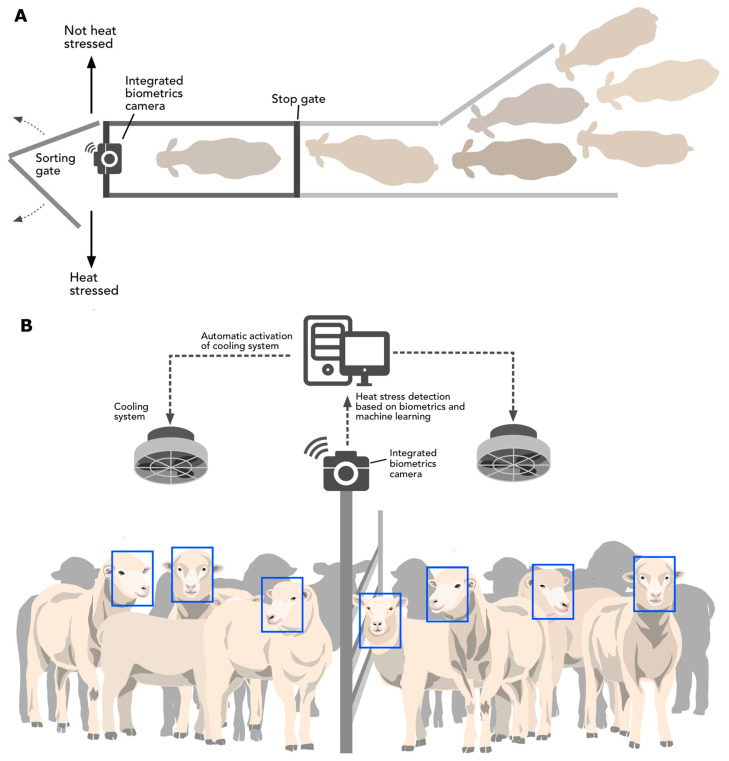
Representation of potential artificial intelligence applications implementing models developed in this study for (**A**) in farm detection of heat-stressed sheep and isolation towards a cooling or shaded area from non-stressed sheep by synchronization with an automated sorting gate, and (**B**) detection of heat stress for sheep in transport coupled with cooling fan systems.

Previously, an AI system to reduce heat stress and increase milk production and quality has been proposed for dairy farms based on the analysis of THI data and cow management information through ML [24]. From the latter study, automatic drafting doors are controlled from ML outputs that transport the cows to the milking area or to a sprinkler-based system to reduce heat stress. A similar method can be implemented here using the algorithms and models developed in this study (Figure 9A). The system proposed has the advantage that there are no physical obstacles between the camera/analysis hub and the individual sheep monitored. In open environments, this AI system could be coupled with virtual fencing systems through collars and the internet of things (IoT) to automate the separation of heat-stressed sheep towards a sprinkler cooling system (Figure 9A). Virtual fencing has been successfully applied for automated cattle control systems [60,61], for sheep [62,63], with acceptable ethical frameworks assessed for their implementation [62,64].

The AI systems proposed can also be implemented in confined sheep in the preparation or during transport (Figure 9B), which could be coupled to blockchain [32,33] to have an unbiased control and independent assessment of animal welfare to be applied in the farm and transport or vessel environments.

## 5. Conclusions

This study proposed the implementation of automated computer vision algorithms and machine learning models to obtain critical biometrics from recorded RGB, and infrared thermal videos from sheep, to help in the automated assessment of heat stress. The implementation of the proposed system requires affordable hardware capabilities, such as the FLIR integrated cameras, which can include dedicated AI micro-processors and blockchain technology. The user-friendly AI system proposed would be able to analyze non-invasive biometrics from sheep in the farm automatically, and through their transport to secure animal welfare, through independent analysis of information incorporating blockchain technology for control purposes. Advances proposed in this paper could offer an AI-based system to monitor animal welfare in farms, and also as a tool to assess animal welfare in transport by land or sea independently, using blockchain. The latter not only could serve governments to audit live animal exports but also the industry in general, for more transparency to the public in their treatment of living animals for human consumption.

## Figures and Tables

**Table 1:** Results of the artificial neural networks pattern recognition model (Model 1) showing the accuracy, error, and performance based on means squared error (MSE) for each stage for the selection of cutoff frequency related to low, medium, and high respiration rate signals from computer vision analysis.

Stage	Samples	Accuracy	Error	Performance (MSE)
**Training**	94	100%	0%	<0.01
**Testing**	40	85%	15%	0.10
**Overall**	134	96%	4%	-

**Table 2:** Results of the artificial neural networks regression model (Model 2) showing statistical data such as correlation coefficient (R), slope, and performance based on means squared error (MSE) for each stage.

Stage	Samples	Observations	R	Slope	Performance (MSE)
**Training**	94	188	0.98	0.94	72
**Testing**	40	80	0.84	0.86	512
**Overall**	134	268	0.94	0.92	-
